# Supplementary material for: Beta Lactams Plus Daptomycin Combination Therapy for Infective Endocarditis: An Italian National Survey (BADAS)
Source: Antibiotics (Basel). 2022 Jan 2;11(1):56. doi: 10.3390/antibiotics11010056 (PMC8773184; doi:10.3390/antibiotics11010056)
Supplement: Supplementary file 1 [file antibiotics-11-00056-s001.zip › antibiotics-1445189-supplementary.pdf]

D1 Il centro ospedaliero in cui presta servizio:

Risposte: 54 Saltate: 1

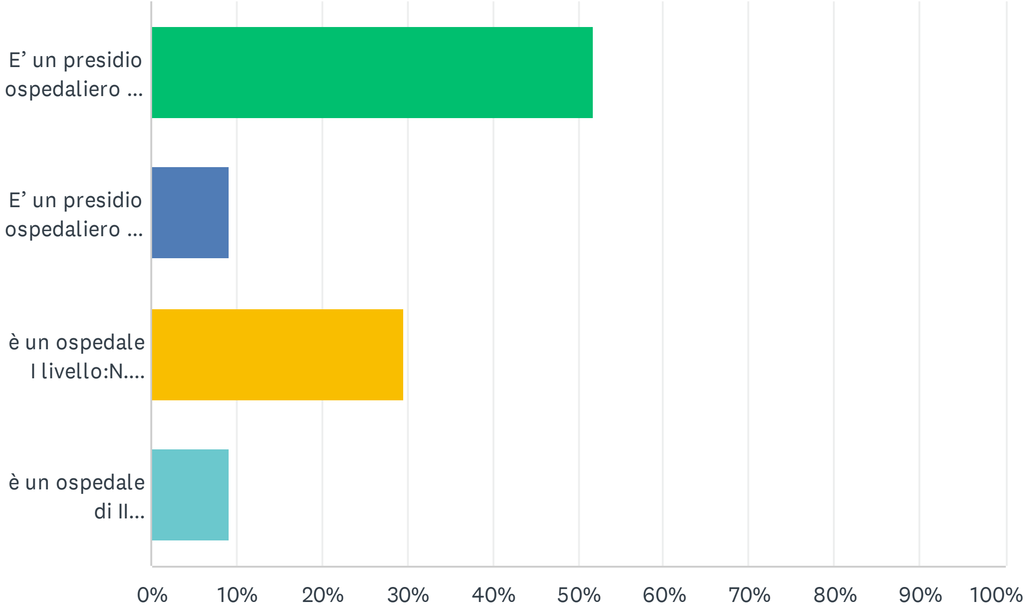

OPZIONI DI RISPOSTA	RISPOSTE	
E’ un presidio ospedaliero o un policlinico universitario	51.85%	28
E’ un presidio ospedaliero di base:N.B. (bacino di utenza 80.000 – 150.000 abitanti), dotati di Pronto Soccorso e delle seguenti specialità: Medicina interna, Chirurgia generale, Ortopedia, Anestesia e servizi di supporto in rete di guardia attiva e/o in regime di pronta disponibilità sulle 24 ore (h.24) di Radiologia, Laboratorio, Emoteca. Devono essere dotati, inoltre, di letti di “Osservazione Breve Intensiva”.	9.26%	5
è un ospedale I livello:N.B. (bacino di utenza 150.000-300.000 abitanti), dotati delle seguenti specialità oltre a quelle presenti nei presidi di base: Ostetricia e Ginecologia (se prevista per numero di parti/anno), Pediatria, Cardiologia con Unità di Terapia Intensiva Cardiologica (U.T.I.C.), Neurologia, Psichiatria, Oncologia, Oculistica, Otorinolaringoiatria, Urologia.	29.63%	16
è un ospedale di II livello:N.B. (bacino di utenza 600.000-1.200.000 abitanti), dotati di DEA di secondo livello e di strutture che attengono anche alle discipline più complesse.	9.26%	5
TOTALE		54

D2 Il centro ospedaliero presso cui svolge la sua attività lavorativa principale conta:

Risposte: 55 Saltate: 0

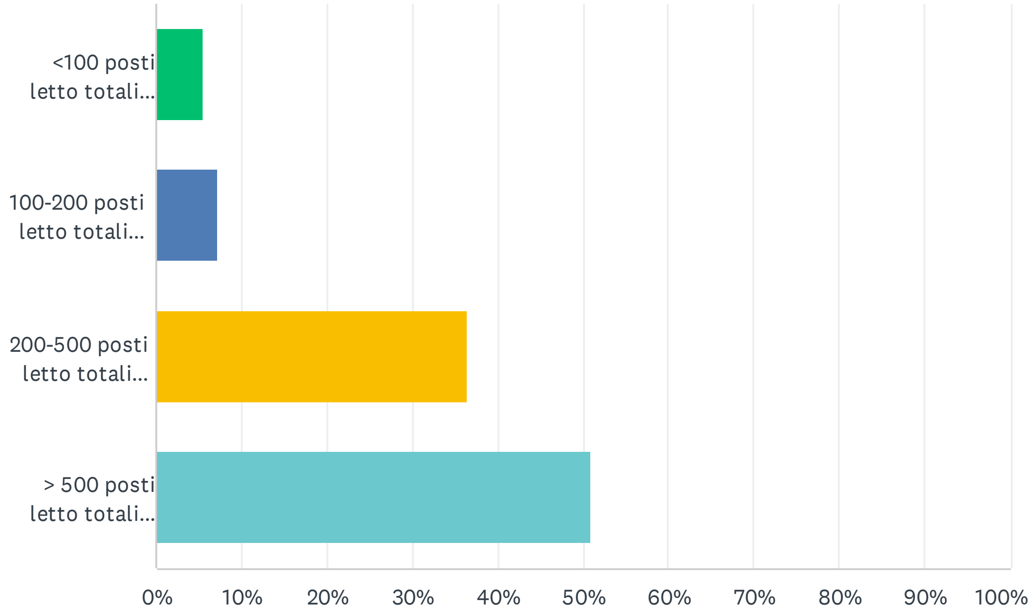

OPZIONI DI RISPOSTA	RISPOSTE	
<100 posti letto totali della struttura	5.45%	3
100-200 posti letto totali della struttura	7.27%	4
200-500 posti letto totali della struttura	36.36%	20
> 500 posti letto totali della struttura	50.91%	28
TOTALE		55

D3 E’ presente ICU nella vostra struttura ospedaliera?

Risposte: 55   Saltate: 0

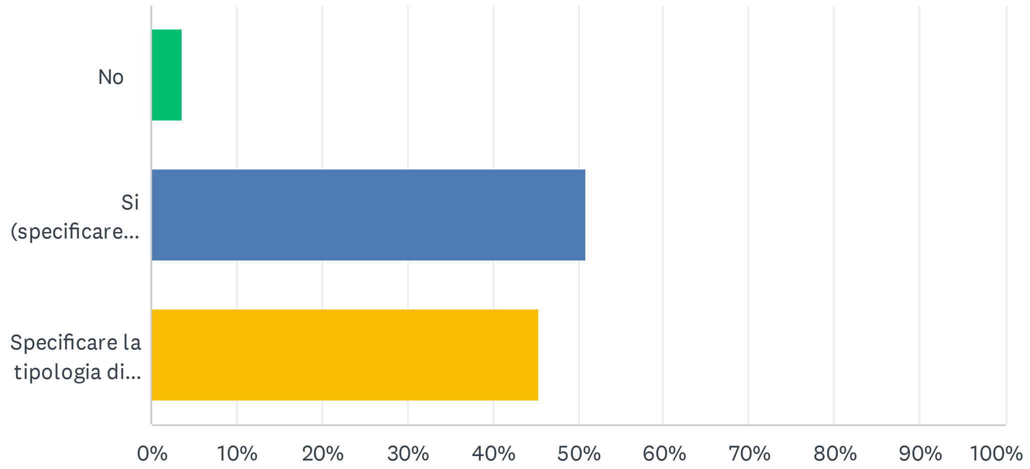

OPZIONI DI RISPOSTA	RISPOSTE	
No	3.64%	2
Si (specificare la tipologia, es. Generale, Respiratoria, Neurologica, Cardio-Toracica, Altra da specificare)	50.91%	28
Specificare la tipologia di ICU	45.45%	25
TOTALE		55

D4 E' presente nella sua struttura ospedaliera il servizio di Unità ad Alta Intensità (High Risk Unit):

Risposte: 54 Saltate: 1

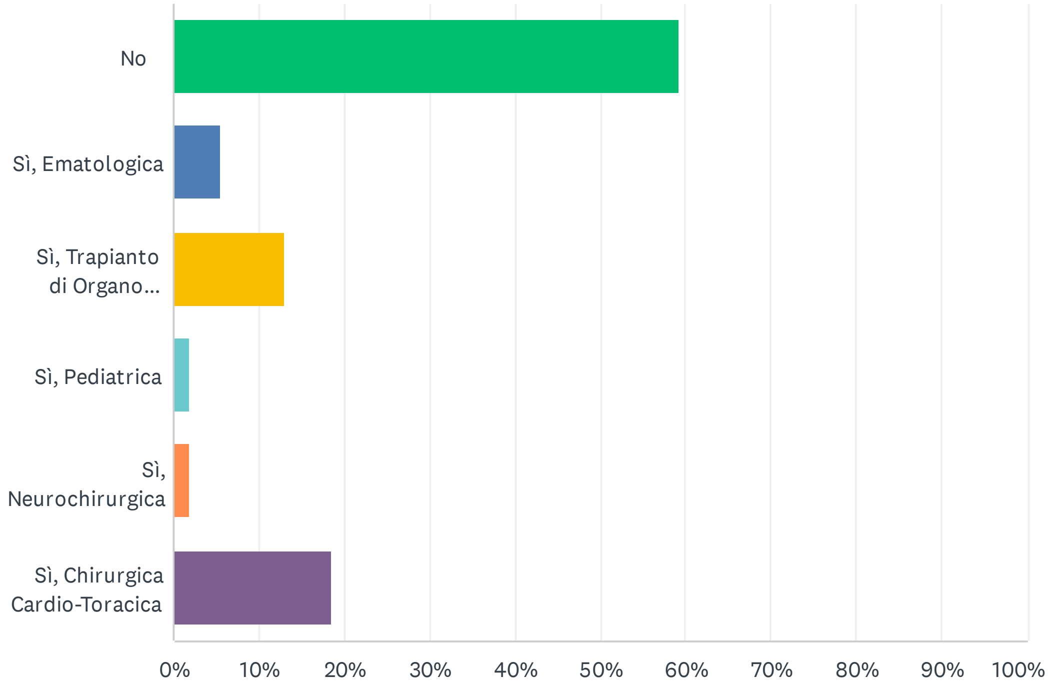

OPZIONI DI RISPOSTA	RISPOSTE	
No	59.26%	32
Sì, Ematologica	5.56%	3
Sì, Trapianto di Organo Solido	12.96%	7
Sì, Pediatrica	1.85%	1
Sì, Neurochirurgica	1.85%	1
Sì, Chirurgica Cardio-Toracica	18.52%	10
TOTALE		54

D5 Nel suo centro, l’infettivologo:

Risposte: 54 Saltate: 1

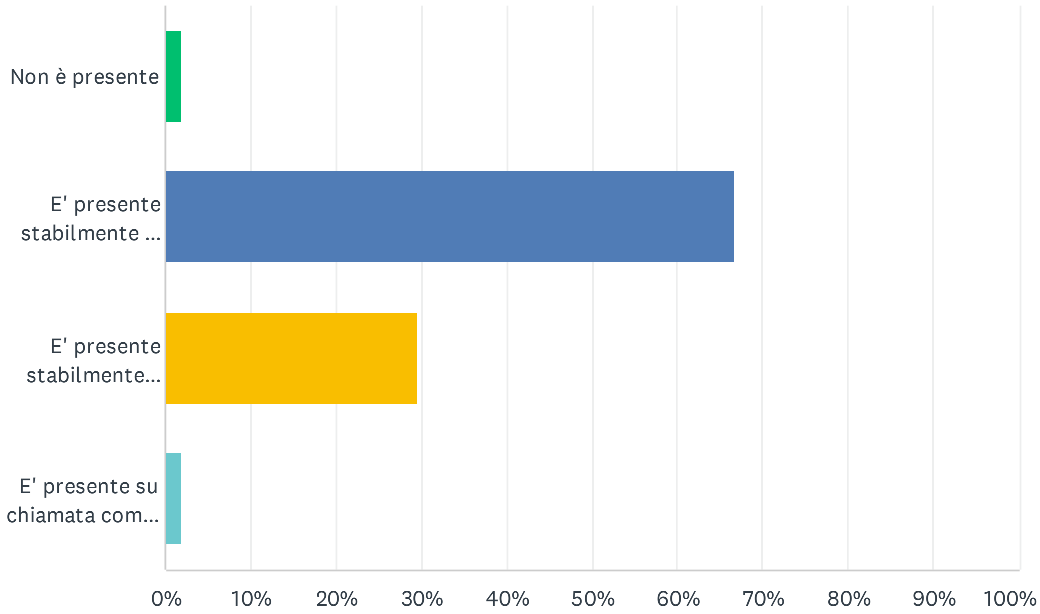

OPZIONI DI RISPOSTA	RISPOSTE	
Non è presente	1.85%	1
E' presente stabilmente e dispone di una struttura di degenza	66.67%	36
E' presente stabilmente nella struttura e dispone di un servizio ambulatoriale e di consulenza	29.63%	16
E' presente su chiamata come consulente proveniente da altra struttura	1.85%	1
TOTALE		54

D6 Sono disponibili nella sua struttura dati epidemiologici riguardanti l'incidenza di infezioni sostenute dai principali organismi multi-resistenti (es. MRSA, MRSE, VRE, CRE...)?

Risposte: 54 Saltate: 1

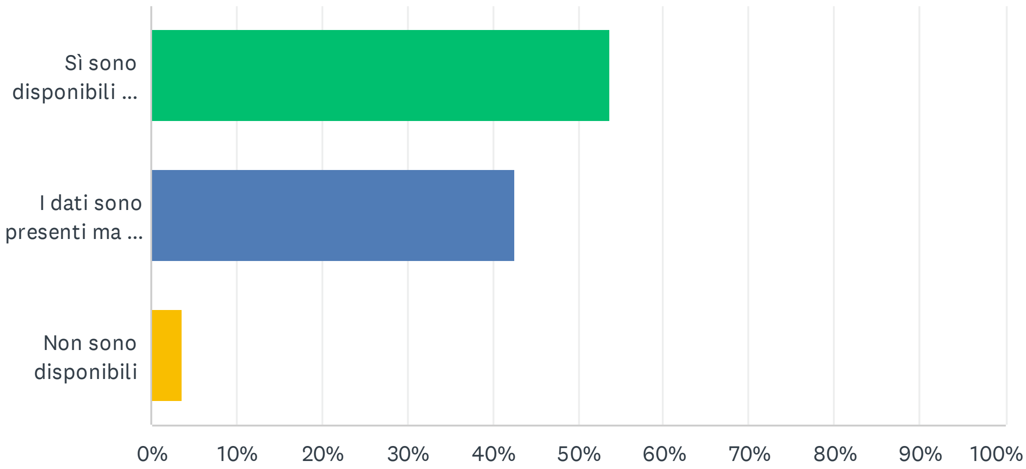

OPZIONI DI RISPOSTA	RISPOSTE	
Sì sono disponibili con incontri annuali	53.70%	29
I dati sono presenti ma non sono facilmente accessibili	42.59%	23
Non sono disponibili	3.70%	2
TOTALE		54

D7 E' presente nella sua struttura un Comitato per il controllo della infezione Ospedaliere (CCI o CIO)?

Risposte: 54 Saltate: 1

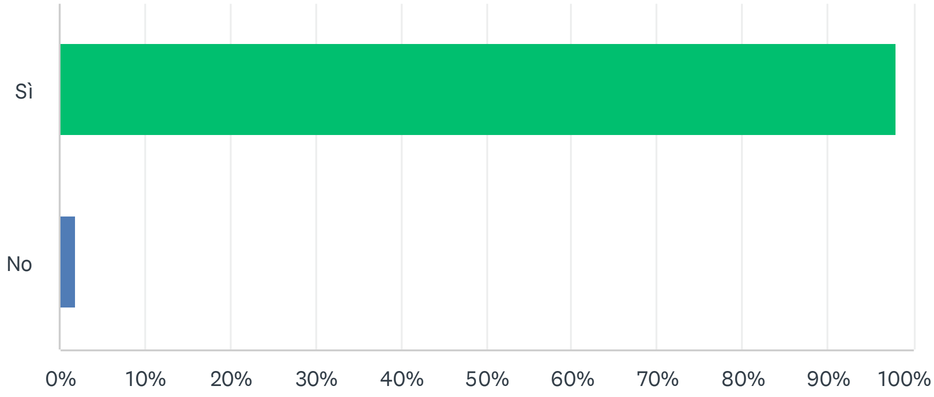

OPZIONI DI RISPOSTA	RISPOSTE	
Sì	98.15%	53
No	1.85%	1
TOTALE		54

D8 Esiste nella sua struttura un sistema di segnalazione rapida delle infezioni correlate all’assistenza (sistema ALERT)?

Risposte: 54 Saltate: 1

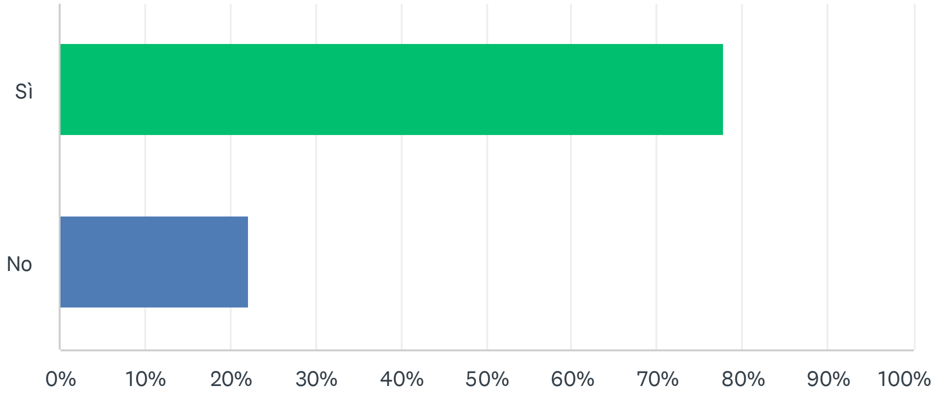

OPZIONI DI RISPOSTA	RISPOSTE	
Sì	77.78%	42
No	22.22%	12
TOTALE		54

D9 Quanti casi di Endocardite Infettive (EI) arrivano alla sua attenzione nell'arco di un anno?

Risposte: 53 Saltate: 2

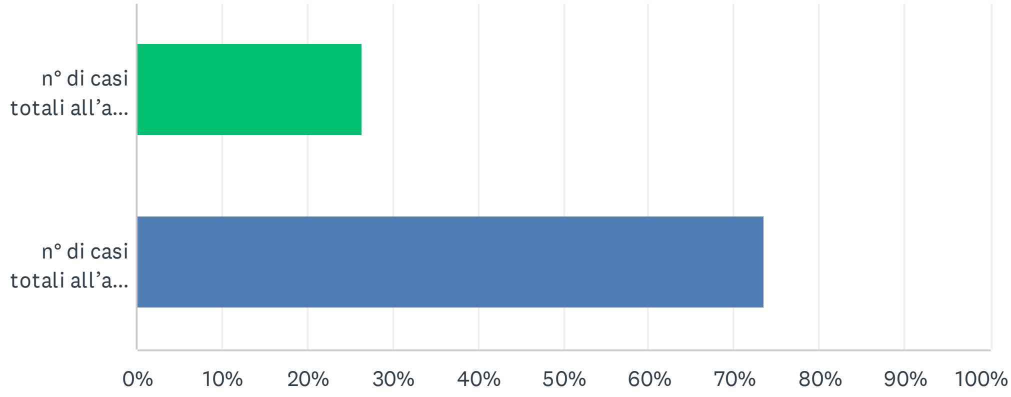

OPZIONI DI RISPOSTA	RISPOSTE	
n° di casi totali all'anno > 50	26.42%	14
n° di casi totali all'anno < 50	73.58%	39
TOTALE		53

D10 Nel suo centro, è presente una struttura di Cardiochirurgia (CCH)?

Risposte: 54   Saltate: 1

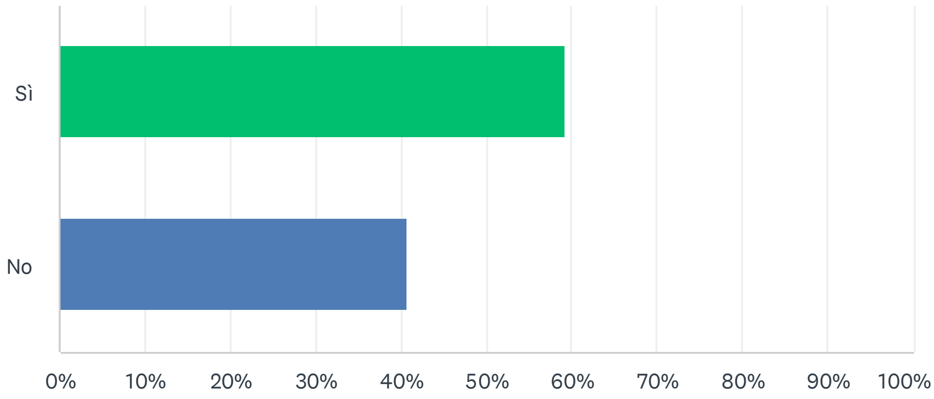

OPZIONI DI RISPOSTA	RISPOSTE	
Sì	59.26%	32
No	40.74%	22
TOTALE		54

D11 Nel suo centro è attivo un gruppo di collaborazione tra Cardiologo, Cardiochirurgo ed Infettivologo per la discussione multidisciplinare dei casi di Endocardite (es. Endocarditis Team)?

Risposte: 53 Saltate: 2

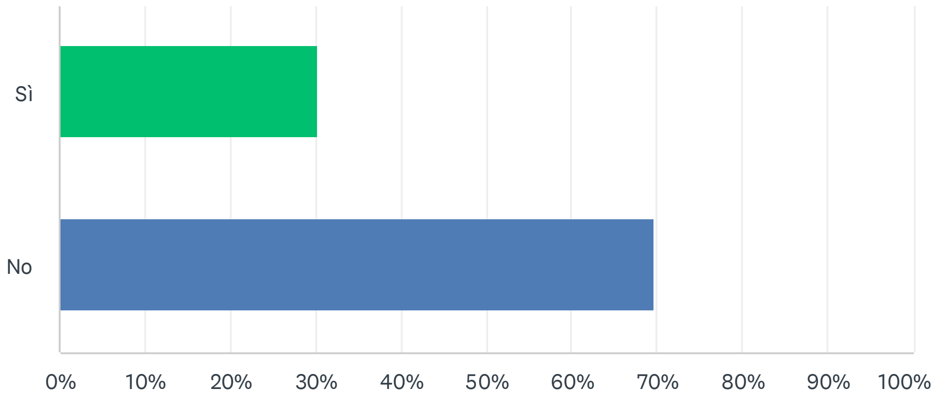

OPZIONI DI RISPOSTA	RISPOSTE	
Sì	30.19%	16
No	69.81%	37
TOTALE		53

D12 Sono disponibili protocolli aziendali/linee guida ospedaliere/linee guida regionali di terapia anti-infettiva?

Risposte: 54 Saltate: 1

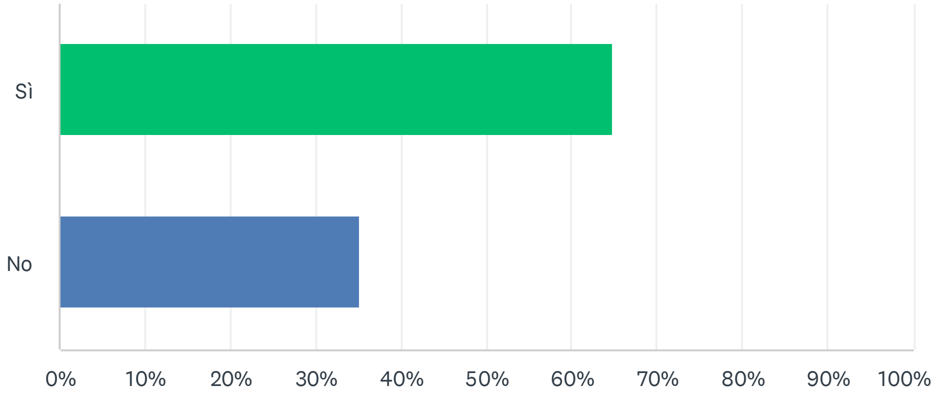

OPZIONI DI RISPOSTA	RISPOSTE	
Sì	64.81%	35
No	35.19%	19
TOTALE		54

D13 Se avete risposto "Sì" alla precedente domanda:

Risposte: 35 Saltate: 20

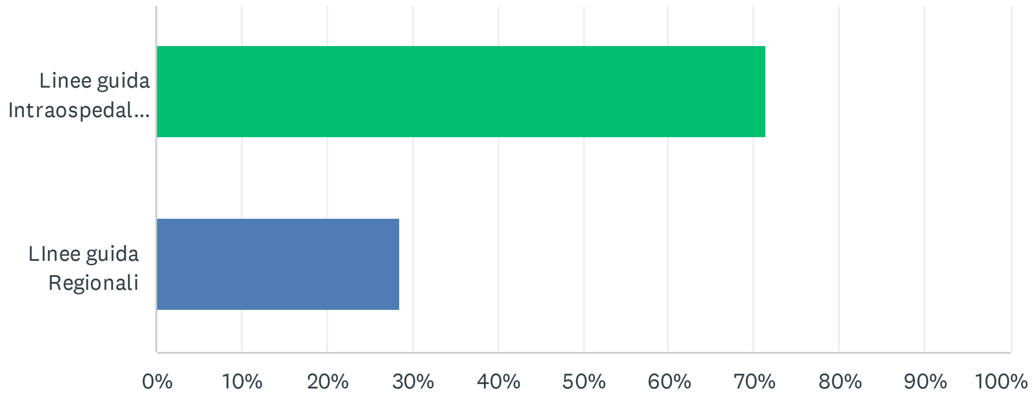

OPZIONI DI RISPOSTA	RISPOSTE	
Linee guida Intraospedaliere	71.43%	25
LInee guida Regionali	28.57%	10
TOTALE		35

D14 Nel sospetto clinico di endocardite infettiva, la terapia antibiotica empirica viene impostata:

Risposte: 54 Saltate: 1

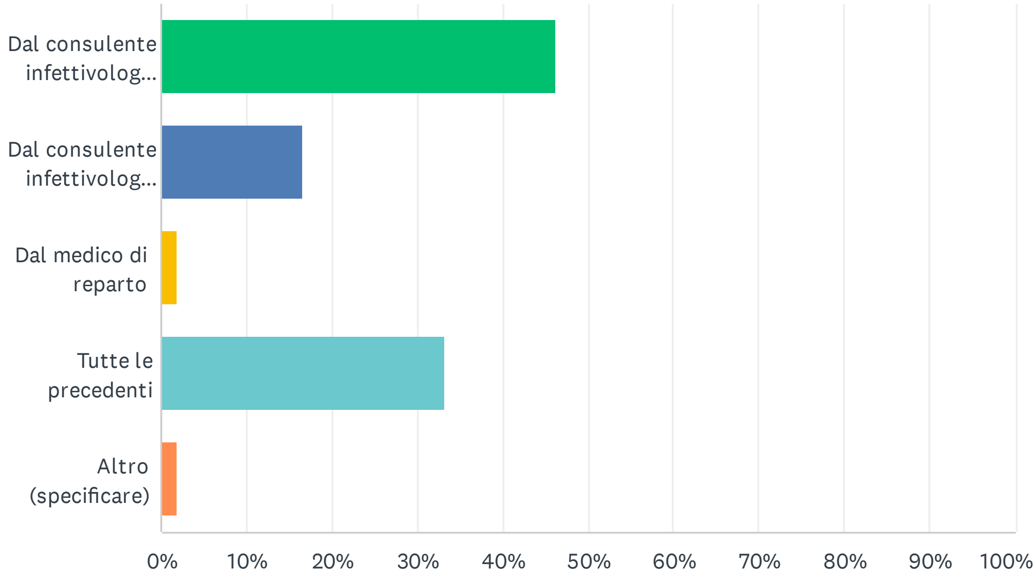

OPZIONI DI RISPOSTA	RISPOSTE	
Dal consulente infettivologo in visita al paziente	46.30%	25
Dal consulente infettivologo anche su consulenza telefonica	16.67%	9
Dal medico di reparto	1.85%	1
Tutte le precedenti	33.33%	18
Altro (specificare)	1.85%	1
TOTALE		54

D15 La terapia empirica dell'EI su valvola nativa (possibili più risposte):

Risposte: 54 Saltate: 1

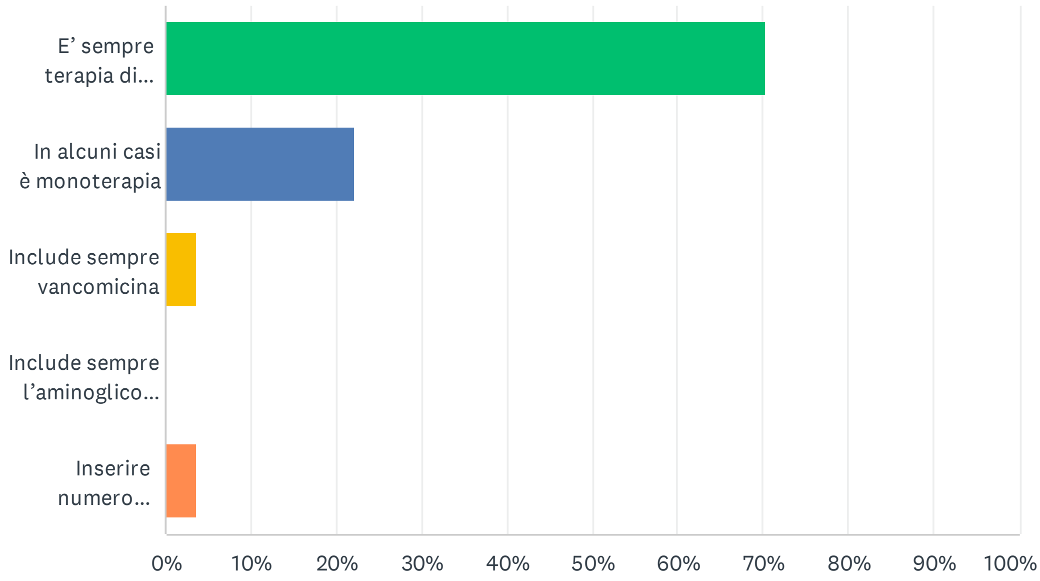

OPZIONI DI RISPOSTA	RISPOSTE	
E' sempre terapia di associazione	70.37%	38
In alcuni casi è monoterapia	22.22%	12
Include sempre vancomicina	3.70%	2
Include sempre l'aminoglicoside	0.00%	0
Inserire numero risposte, se scelta multipla	3.70%	2
TOTALE		54

D16 La terapia empirica dell'EI su valvola protesica (possibili più risposte):

Risposte: 54   Saltate: 1

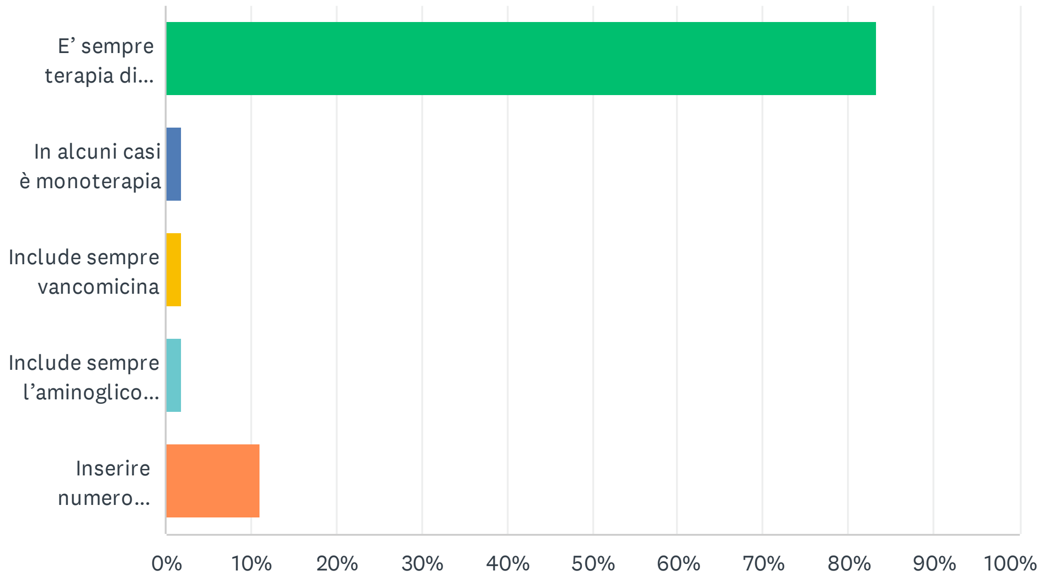

OPZIONI DI RISPOSTA	RISPOSTE	
E' sempre terapia di associazione	83.33%	45
In alcuni casi è monoterapia	1.85%	1
Include sempre vancomicina	1.85%	1
Include sempre l'aminoglicoside	1.85%	1
Inserire numero risposte, se scelta multipla	11.11%	6
TOTALE		54

D17 In media la durata della terapia per EI non sottoposta a trattameno chirugico :

Risposte: 54 Saltate: 1

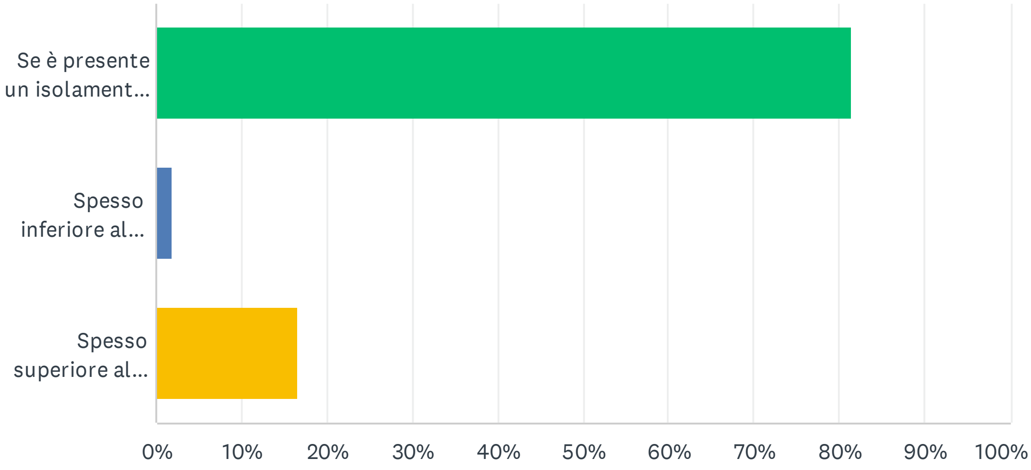

OPZIONI DI RISPOSTA	RISPOSTE	
Se è presente un isolamento su emocoltura viene stabilita in base all' agente eziologico con un range compreso, nella maggior parte dei casi, tra le 2-6 settimane	81.48%	44
Spesso inferiore alle 6 settimane, indipendentemente dall' isolamento	1.85%	1
Spesso superiore alle 6 settimane, indipendentemente dall' isolamento	16.67%	9
TOTALE		54

D18 In media la durata della terapia per EI sottoposta a trattamento chirurgico:

Risposte: 54 Saltate: 1

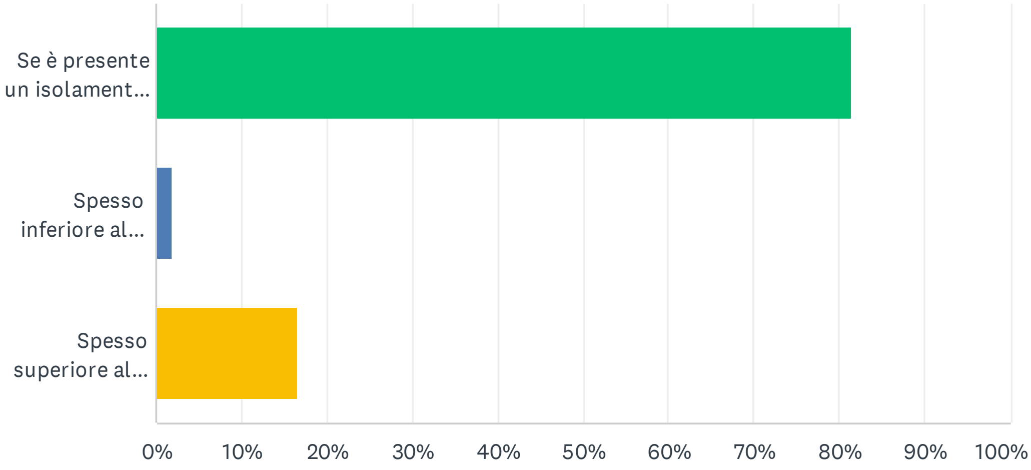

OPZIONI DI RISPOSTA	RISPOSTE	
Se è presente un isolamento su emocoltura viene stabilita in base all’agente eziologico con un range compreso, nella maggior parte dei casi, tra le 2-6 settimane	81.48%	44
Spesso inferiore alle 6 settimane, indipendentemente dall’isolamento	1.85%	1
Spesso superiore alle 6 settimane, indipendentemente dall’isolamento	16.67%	9
TOTALE		54

D19 Qual è la durata media della terapia dell'EI nei diversi setting clinici (inserire durata in settimane):

Risposte: 55 Saltate: 0

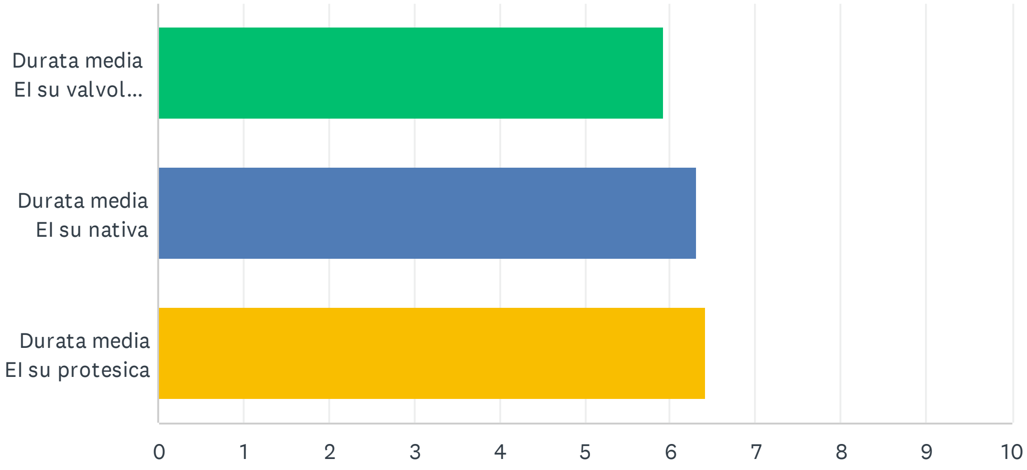

OPZIONI DI RISPOSTA	NUMERO MEDIO	NUMERO TOTALE	RISPOSTE
Durata media EI su valvola non operata	6	326	55
Durata media EI su nativa	6	347	55
Durata media EI su protesica	6	353	55
Totale rispondenti: 55			

STATISTICHE DI BASE					
	MINIMO	MASSIMO	MEDIANA	MEDIA	DEVIAZIONE STANDARD
Durata media EI su protesica	4.00	12.00	6.00	6.42	1.46
Durata media EI su valvola non operata	3.00	20.00	6.00	5.93	2.19
Durata media EI su nativa	2.00	89.00	4.00	6.31	11.31

D20 La confidenza nella sua attività clinica nell'uso di daptomicina in empirico è maggiore per (più risposte possibili):

Risposte: 52 Saltate: 3

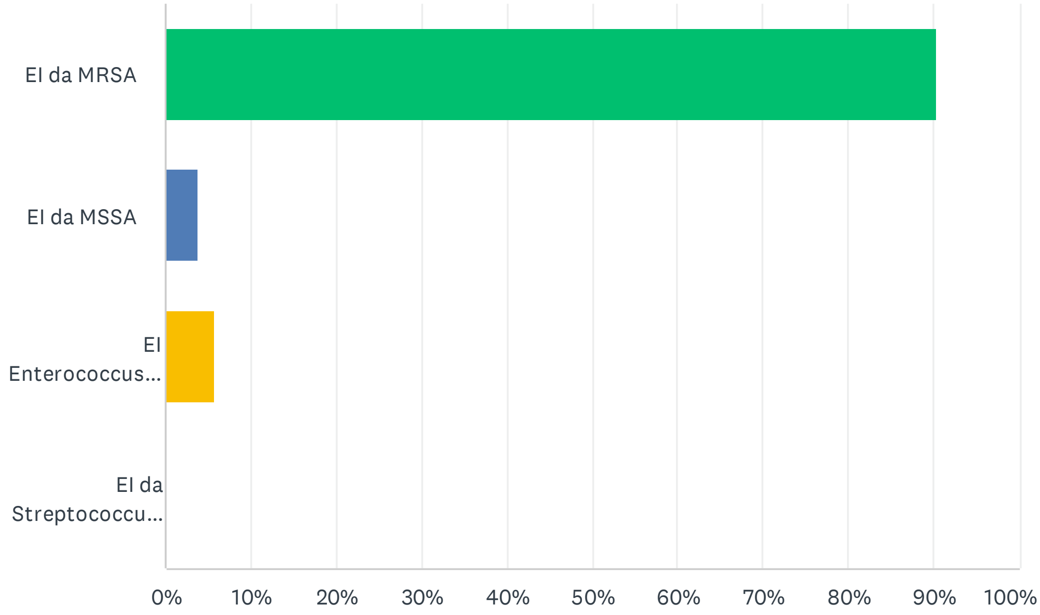

OPZIONI DI RISPOSTA	RISPOSTE	
EI da MRSA	90.38%	47
EI da MSSA	3.85%	2
EI Enterococcus spp.	5.77%	3
EI da Streptococcus spp.	0.00%	0
TOTALE		52

D21 Nel caso di un paziente con endocardite su valvola mitralica nativa da Streptococcus sanguis con MIC alla penicillina ≤0.125 mg/l, generalmente il trattamento:

Risposte: 52 Saltate: 3

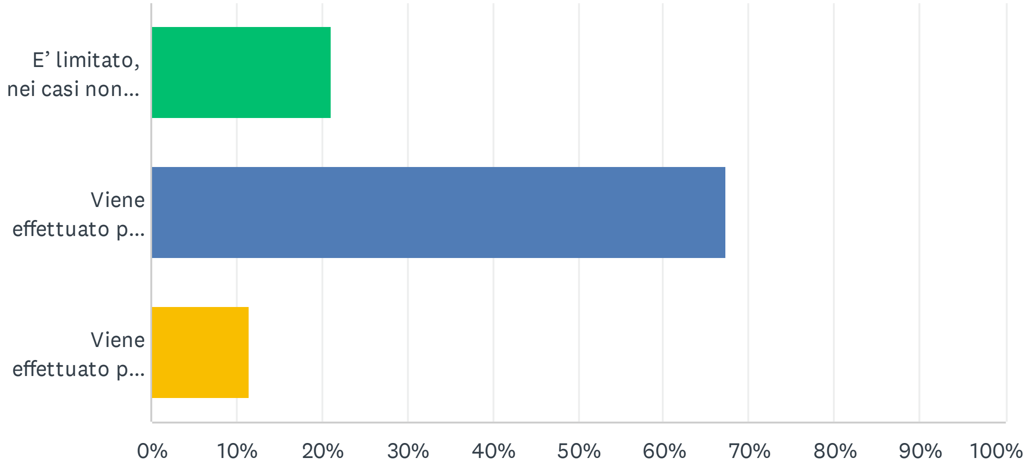

OPZIONI DI RISPOSTA	RISPOSTE	
E' limitato, nei casi non complicati, a 2 settimane	21.15%	11
Viene effettuato per 4 settimane	67.31%	35
Viene effettuato per più di 4 settimane	11.54%	6
TOTALE		52

D22 Nel caso di un paziente con endocardite su valvola protesica da Staphylococcus aureus MS, generalmente il trattamento viene effettuato:

Risposte: 53 Saltate: 2

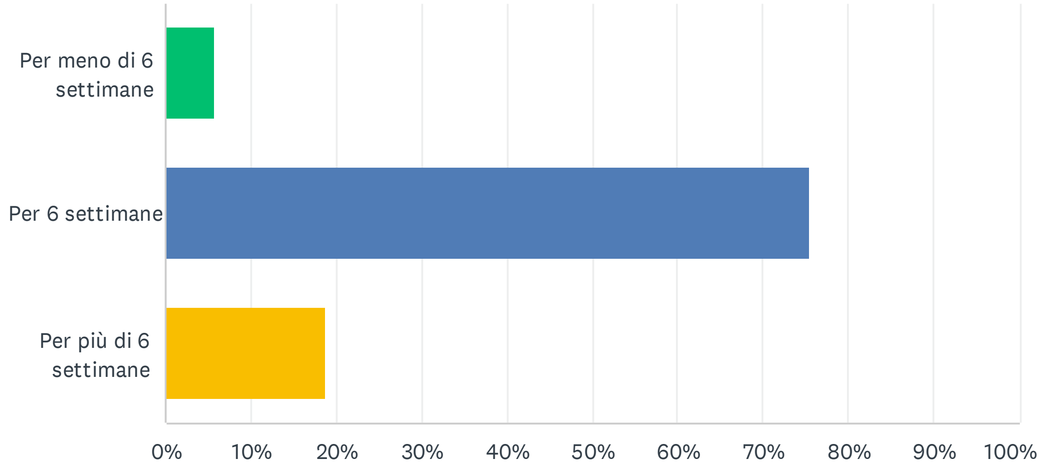

OPZIONI DI RISPOSTA	RISPOSTE	
Per meno di 6 settimane	5.66%	3
Per 6 settimane	75.47%	40
Per più di 6 settimane	18.87%	10
TOTALE		53

D23 Ha avuto nel suo centro episodi di resistenza in corso di Daptomicina nel trattamento di endocarditi infettive da Streptococcus spp.

Risposte: 54 Saltate: 1

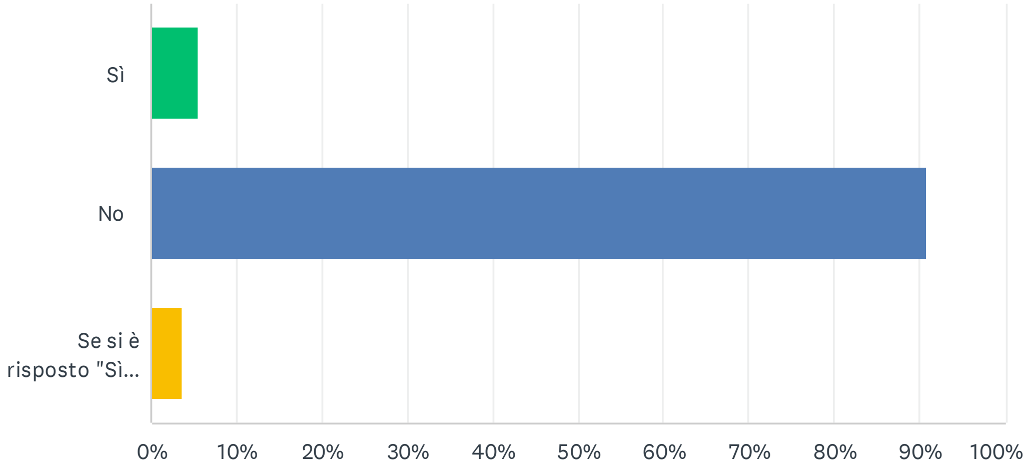

OPZIONI DI RISPOSTA	RISPOSTE	
Sì	5.56%	3
No	90.74%	49
Se si è risposto "Sì", di quanti episodi è a conoscenza nel biennio 2018-2019	3.70%	2
TOTALE		54

D24 Se sono stati riscontrati casi di resistenza a Daptomicina in corsi di EI da Streptococcus spp., questi si sono verificati in corso di:

Risposte: 12 Saltate: 43

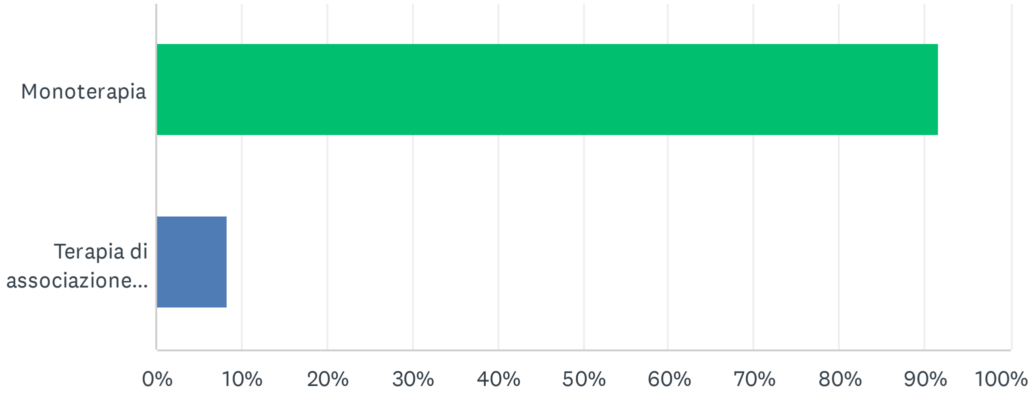

OPZIONI DI RISPOSTA	RISPOSTE	
Monoterapia	91.67%	11
Terapia di associazione (es. Daptomicina + Cefalosporina)	8.33%	1
TOTALE		12

D25 Nel caso di un paziente con endocardite su valvola protesica da Staphylococcus aureus MR, generalmente il trattamento viene effettuato:

Risposte: 53 Saltate: 2

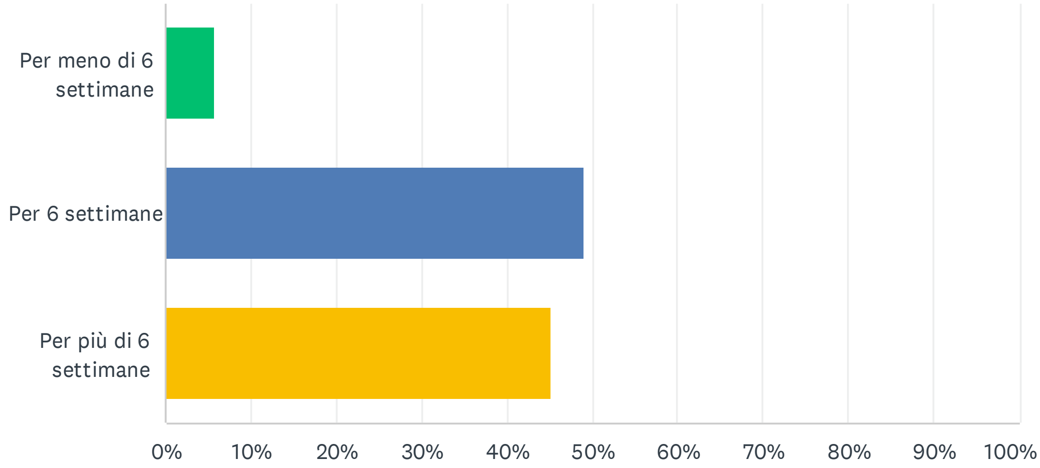

OPZIONI DI RISPOSTA	RISPOSTE	
Per meno di 6 settimane	5.66%	3
Per 6 settimane	49.06%	26
Per più di 6 settimane	45.28%	24
TOTALE		53

D26 Nel caso di un paziente con endocardite su valvola protesica da Enterococcus spp., generalmente il trattamento viene effettuato:

Risposte: 53 Saltate: 2

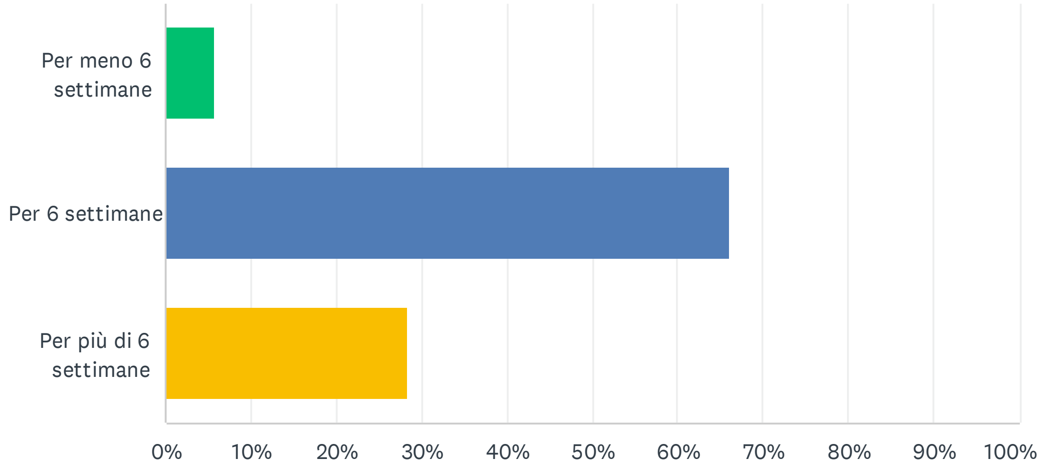

OPZIONI DI RISPOSTA	RISPOSTE	
Per meno 6 settimane	5.66%	3
Per 6 settimane	66.04%	35
Per più di 6 settimane	28.30%	15
TOTALE		53

D27 Relativamente all’utilizzo della daptomicina, nella sua attività clinica:

Risposte: 54   Saltate: 1

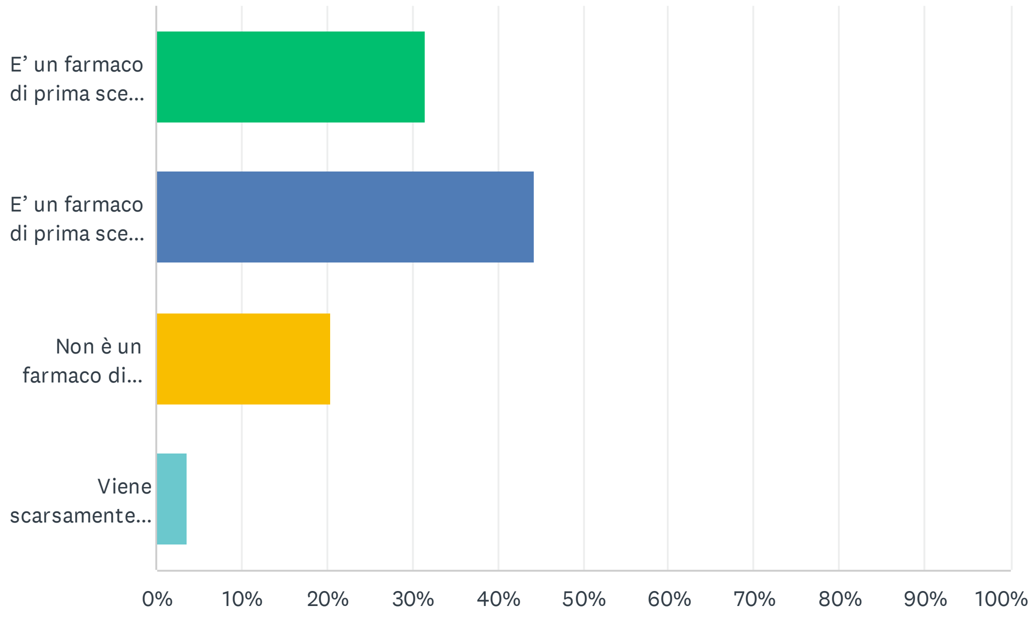

OPZIONI DI RISPOSTA	RISPOSTE	
E’ un farmaco di prima scelta per la terapia empirica	31.48%	17
E’ un farmaco di prima scelta per la terapia mirata delle endocarditi da MRSA	44.44%	24
Non è un farmaco di prima scelta	20.37%	11
Viene scarsamente utilizzata nel trattamento delle endocarditi infettive (<15% dei casi)	3.70%	2
TOTALE		54

D28 In caso di utilizzo di daptomicina, generalmente la motivazione principale della sua scelta in terapia empirica per endocarditi su valvola nativa è (possibili risposte multiple):

Risposte: 53   Saltate: 2

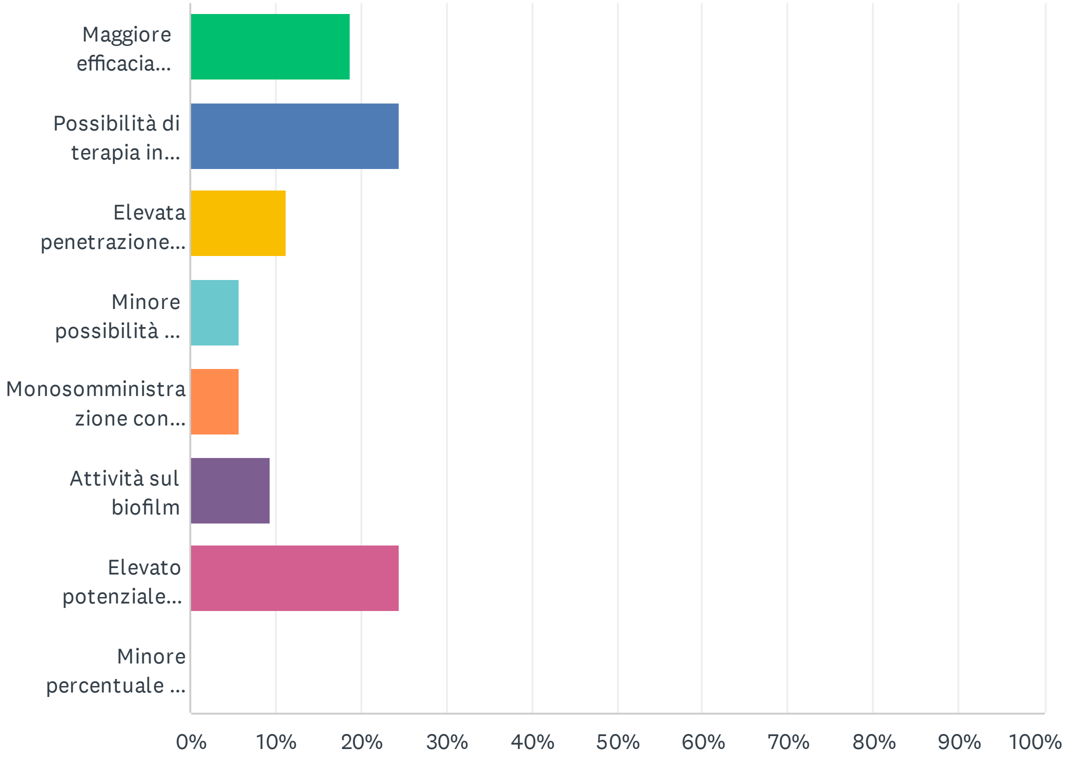

OPZIONI DI RISPOSTA	RISPOSTE	
Maggiore efficacia clinica	18.87%	10
Possibilità di terapia in associazione con regime aminoglicoside-sparing	24.53%	13
Elevata penetrazione nelle vegetazioni	11.32%	6
Minore possibilità di eventi avversi/tossicità	5.66%	3
Monosomministrazione con conseguente possibilità di prosecuzione della terapia endovenosa post-dimissione	5.66%	3
Attività sul biofilm	9.43%	5
Elevato potenziale battericida	24.53%	13
Minore percentuale di ceppi Daptomicina-R e/o MIC più vantaggiose	0.00%	0
TOTALE		53

D29 In caso di utilizzo di daptomicina, generalmente la motivazione principale della sua scelta in terapia empirica per endocarditi su valvola protesica è (possibili risposte multiple):

Risposte: 51 Saltate: 4

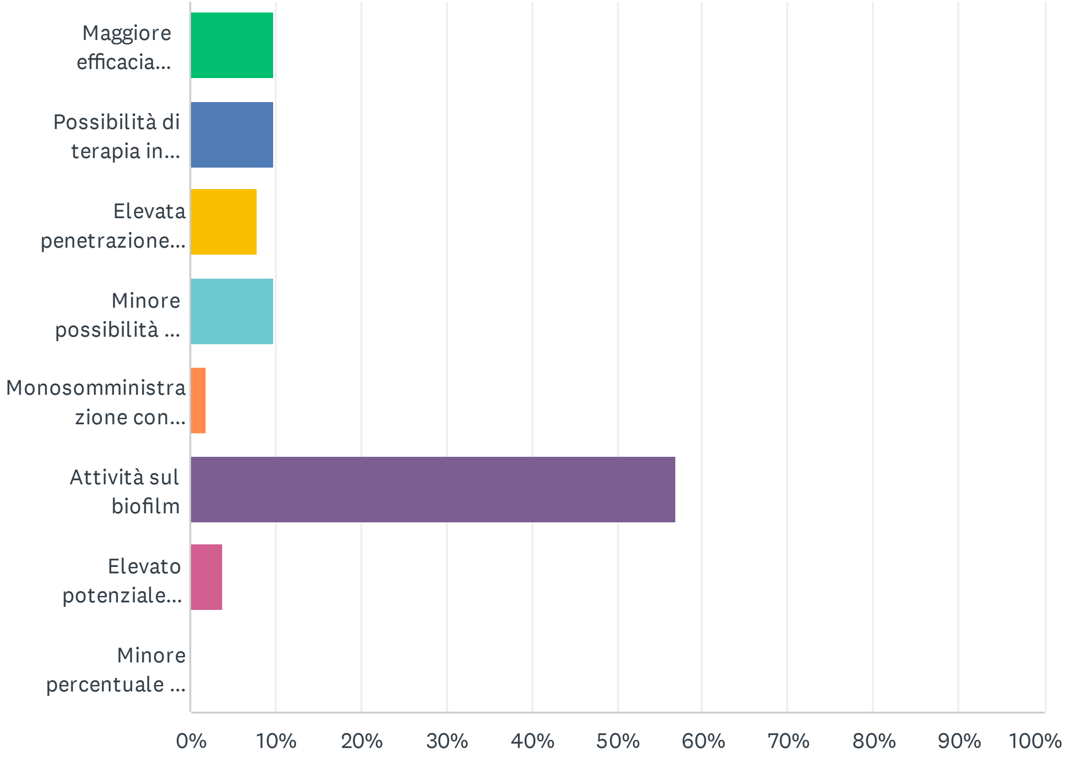

OPZIONI DI RISPOSTA	RISPOSTE	
Maggiore efficacia clinica	9.80%	5
Possibilità di terapia in associazione con regime aminoglicoside-sparing	9.80%	5
Elevata penetrazione nelle vegetazioni	7.84%	4
Minore possibilità di eventi avversi/tossicità	9.80%	5
Monosomministrazione con conseguente possibilità di prosecuzione della terapia endovenosa post-dimissione	1.96%	1
Attività sul biofilm	56.86%	29
Elevato potenziale battericida	3.92%	2
Minore percentuale di ceppi Daptomicina-R e/o MIC più vantaggiose	0.00%	0
TOTALE		51

D30 In caso di utilizzo di daptomicina, generalmente nella sua attività clinica preferisce:

Risposte: 54 Saltate: 1

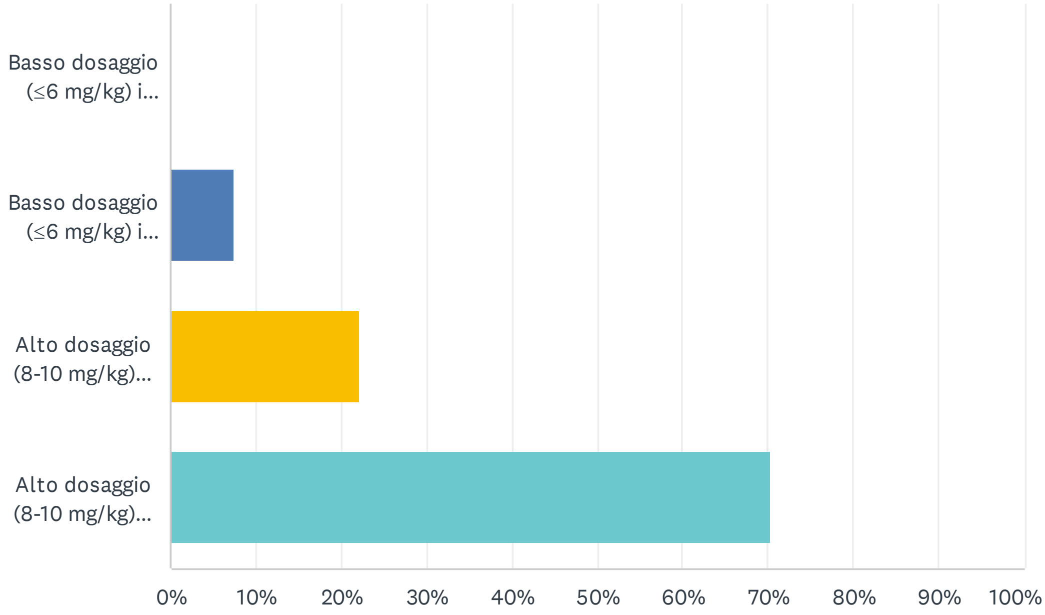

OPZIONI DI RISPOSTA	RISPOSTE	
Basso dosaggio (≤6 mg/kg) in monoterapia	0.00%	0
Basso dosaggio (≤6 mg/kg) in associazione	7.41%	4
Alto dosaggio (8-10 mg/kg) in monoterapia	22.22%	12
Alto dosaggio (8-10 mg/kg) in associazione	70.37%	38
TOTALE		54

D31 In caso di utilizzo di daptomicina in associazione, generalmente nella sua attività clinica, preferisce come farmaco partner:

Risposte: 48   Saltate: 7

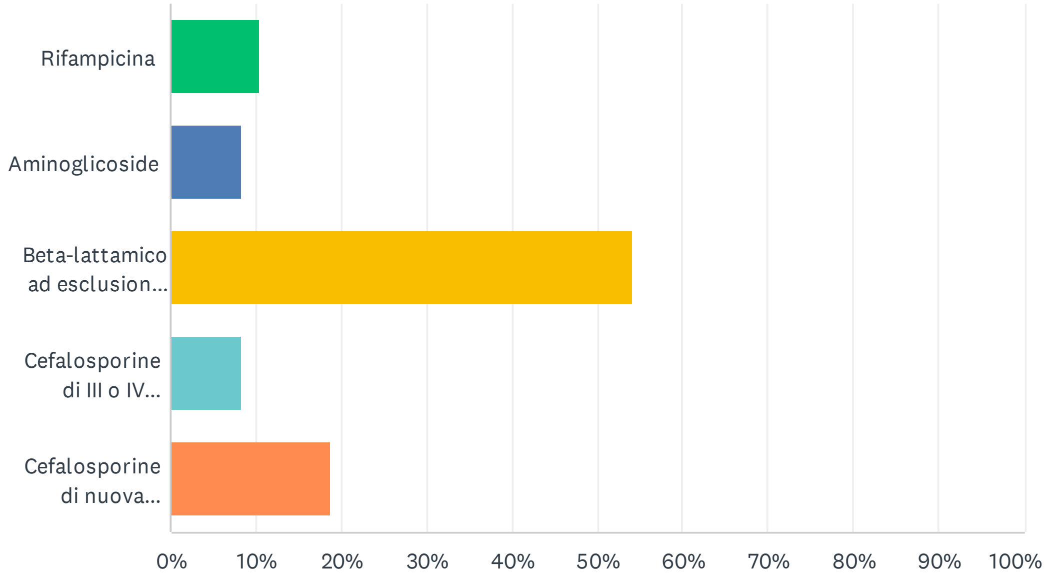

OPZIONI DI RISPOSTA	RISPOSTE	
Rifampicina	10.42%	5
Aminoglicoside	8.33%	4
Beta-lattamico ad esclusione delle cefalosporine di nuova generazione	54.17%	26
Cefalosporine di III o IV generazione	8.33%	4
Cefalosporine di nuova generazione (es: ceftarolina, ceftobiprole)	18.75%	9
TOTALE		48

D32 In caso di utilizzo di daptomicina, questo avviene più comunemente per la terapia mirata di endocarditi da:

Risposte: 54 Saltate: 1

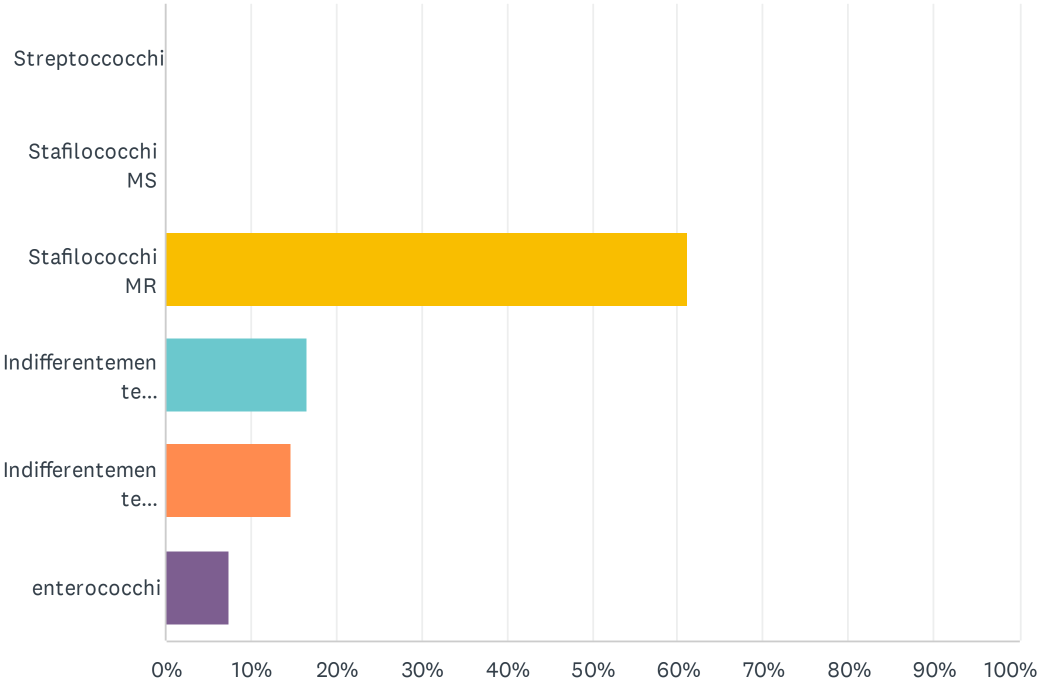

OPZIONI DI RISPOSTA	RISPOSTE	
Streptoccocchi	0.00%	0
Stafilococchi MS	0.00%	0
Stafilococchi MR	61.11%	33
Indifferentemente stafilococchi MS e MR	16.67%	9
Indifferentemente streptococchi e stafilococchi MS e MR	14.81%	8
enterococchi	7.41%	4
TOTALE		54

D33 L'associazione daptomicina + beta-lattamico nella sua attività clinica viene più comunemente utilizzata per la terapia mirata delle endocarditi da:

Risposte: 54 Saltate: 1

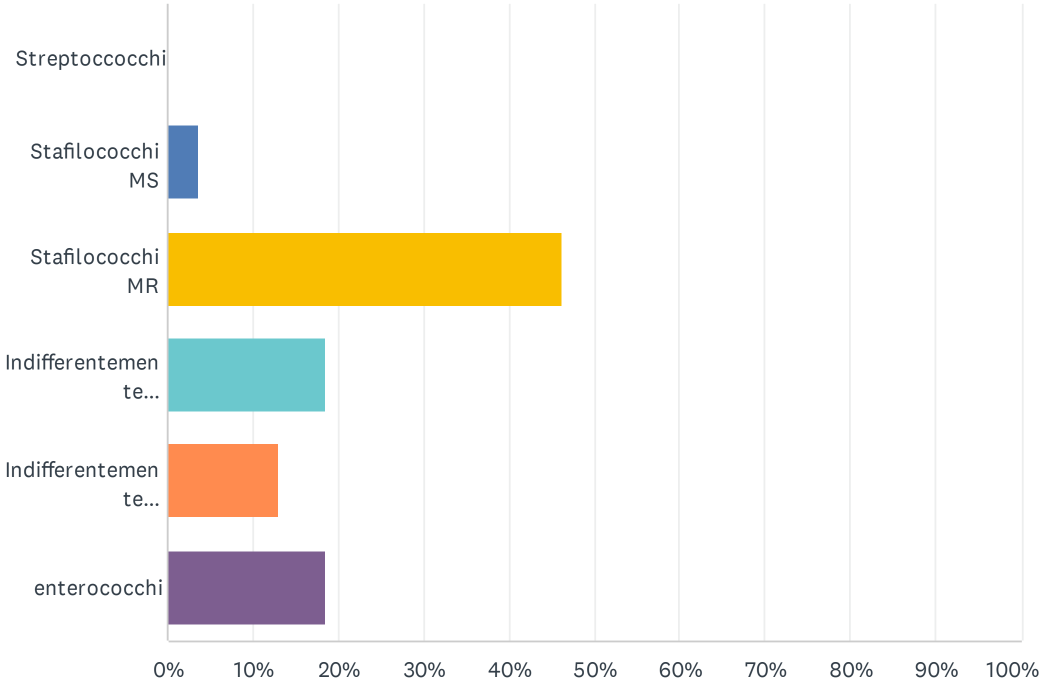

OPZIONI DI RISPOSTA	RISPOSTE	
Streptoccocchi	0.00%	0
Stafilococchi MS	3.70%	2
Stafilococchi MR	46.30%	25
Indifferentemente stafilococchi MS e MR	18.52%	10
Indifferentemente streptococchi e stafilococchi MS e MR	12.96%	7
enterococchi	18.52%	10
TOTALE		54

D34 In caso di utilizzo dell’associazione daptomicina + beta-lattamico, questa è più comunemente utilizzata:

Risposte: 53 Saltate: 2

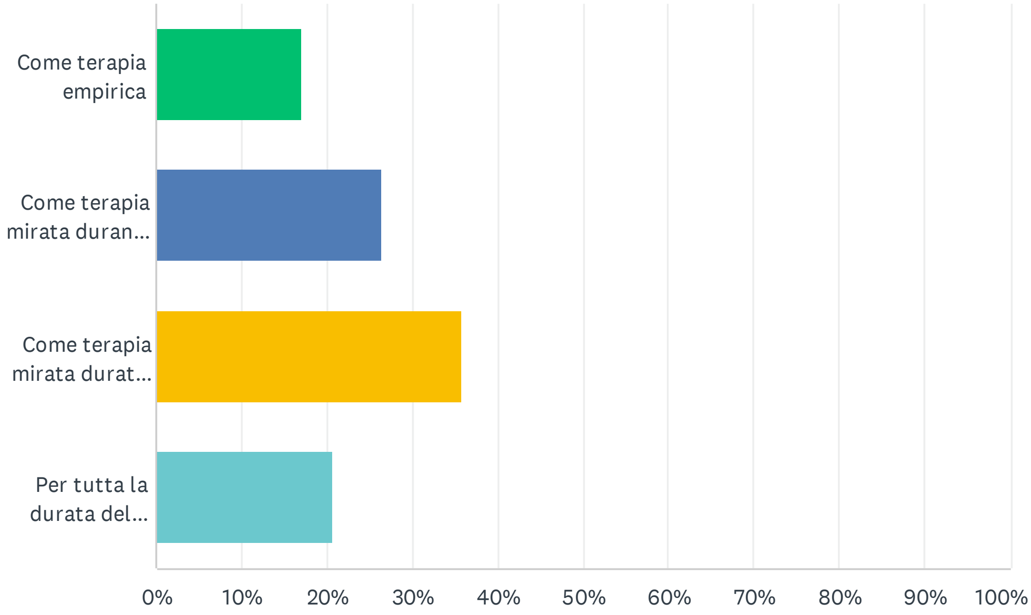

OPZIONI DI RISPOSTA	RISPOSTE	
Come terapia empirica	16.98%	9
Come terapia mirata durante l’ospedalizzazione e poi sottoposta a semplificazione con interruzione del beta-lattamico e prosecuzione della daptomicina in post-dimissione	26.42%	14
Come terapia mirata durata l’ospedalizzazione e poi sottoposta a semplificazione con sostituzione con terapia per os	35.85%	19
Per tutta la durata del trattamento in regime di ospedalizzazione	20.75%	11
TOTALE		53

D35 Nel paziente stabile/operato in terapia mirata con daptomicina in associazione dopo quanti giorni di terapia si può pensare ad una de-escalation (monoterapia, switch per os, sospensione):

Risposte: 55 Saltate: 0

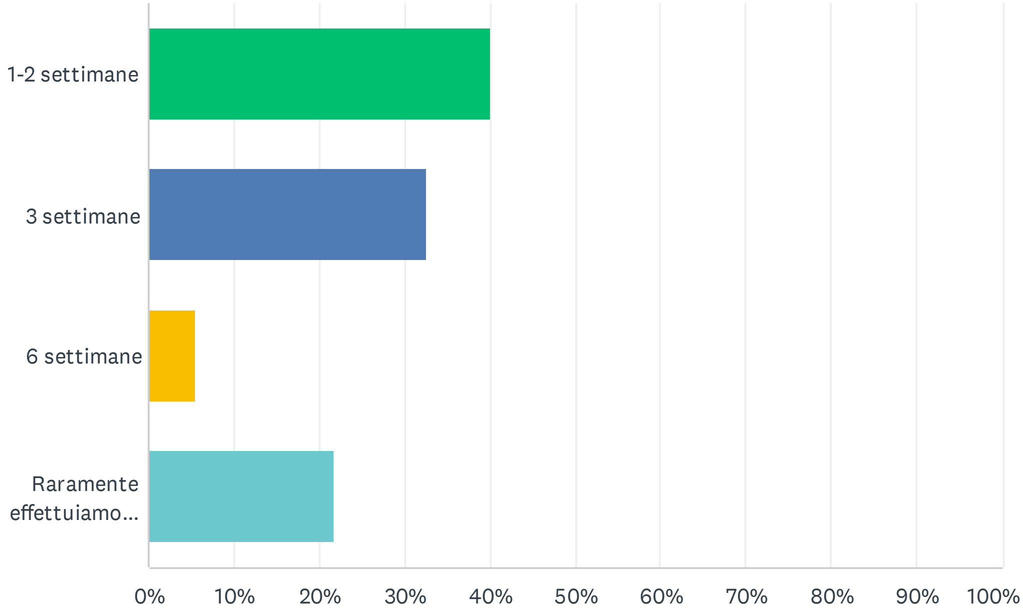

OPZIONI DI RISPOSTA	RISPOSTE	
1-2 settimane	40.00%	22
3 settimane	32.73%	18
6 settimane	5.45%	3
Raramente effettuiamo de-escalation nel nostro centro	21.82%	12
TOTALE		55

D36 Quali sono i farmaci utilizzati per la de-escalation (possibile più di un farmaco):

Risposte: 55   Saltate: 0

D37 La sostituzione in terapia di Daptomicina con un’altra molecola, avviene più frequentemente a causa di:

Risposte: 54 Saltate: 1

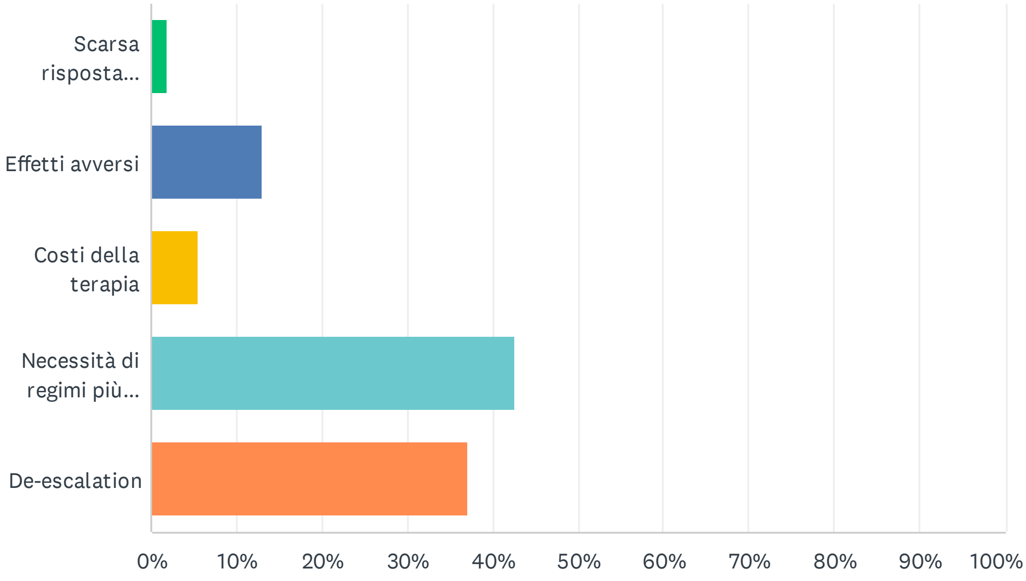

OPZIONI DI RISPOSTA	RISPOSTE	
Scarsa risposta clinica e laboratoristica	1.85%	1
Effetti avversi	12.96%	7
Costi della terapia	5.56%	3
Necessità di regimi più facilmente gestibili in regimi di lungodegenza o cure domiciliari	42.59%	23
De-escalation	37.04%	20
TOTALE		54

D38 Vi chiediamo infine la specialità medica o il ruolo che ricoprite nel vostro ospedale:

Risposte: 54 Saltate: 1

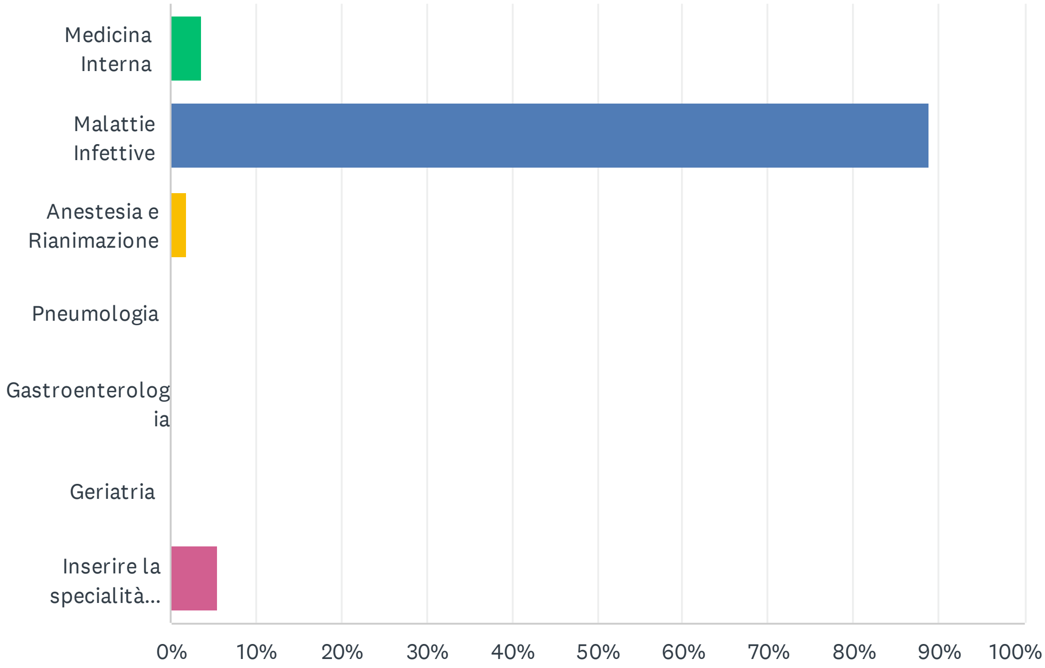

OPZIONI DI RISPOSTA	RISPOSTE	
Medicina Interna	3.70%	2
Malattie Infettive	88.89%	48
Anestesia e Rianimazione	1.85%	1
Pneumologia	0.00%	0
Gastroenterologia	0.00%	0
Geriatria	0.00%	0
Inserire la specialità medica o il ruolo che ricoprite nel vostro centro, se altro	5.56%	3
TOTALE		54

D39 Vuole che il suo nominativo venga inserito, in qualità di collaboratore, all'interno della nostra survey?

Risposte: 53 Saltate: 2

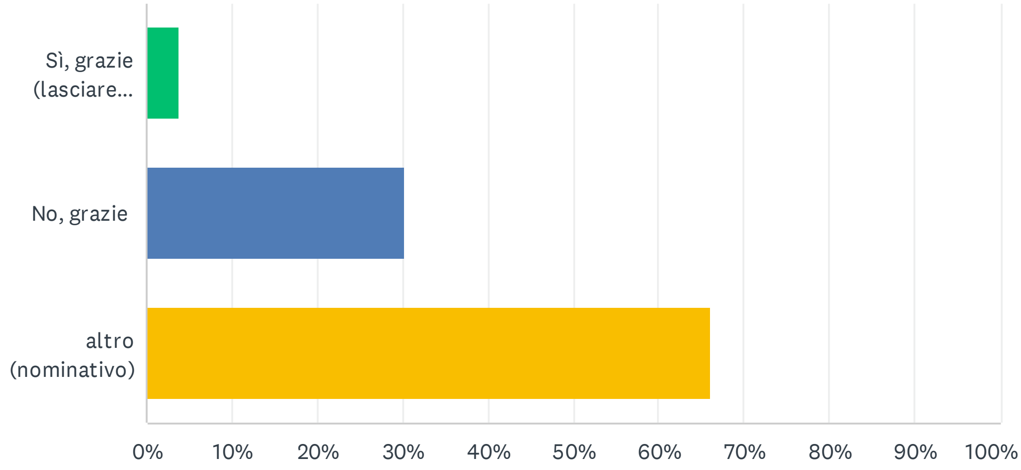

OPZIONI DI RISPOSTA	RISPOSTE	
Sì, grazie (lasciare gentilmente il nominativo nel campo "altro")	3.77%	2
No, grazie	30.19%	16
altro (nominativo)	66.04%	35
TOTALE		53
